# Characteristics and spectrum changes of PICU cases during the COVID-19 pandemic: a retrospective analysis

**DOI:** 10.3389/fped.2024.1325471

**Published:** 2024-04-25

**Authors:** Yufan Yang, Yueqi He, Jiaotian Huang, Haipeng Yan, Xinping Zhang, Zhenghui Xiao, Xiulan Lu

**Affiliations:** Department of Intensive Care Unit, Affiliated School of Medicine of Central South University (Hunan Children's Hospital), Changsha, Hunan, China

**Keywords:** COVID-19 pandemic, PICU, disease spectrum, children, spectrum changes

## Abstract

**Objective:**

This study aims to compare the changes in the disease spectrum of children admitted to the Pediatric Intensive Care Units (PICU) during the COVID-19 pandemic with the three years prior to the pandemic, exploring the impact of the COVID-19 pandemic on the disease spectrum of PICU patients.

**Methods:**

A retrospective analysis was conducted on critically ill children admitted to the PICU of Hunan Children's Hospital from January 2020 to December 2022, and the results were compared with cases from the same period between January 2017 and December 2019. The cases were divided into pre-pandemic period (January 2017–December 2019) with 8,218 cases, and pandemic period (January 2020–December 2022) with 5,619 cases. General characteristics, age, and gender were compared between the two groups.

**Results:**

Compared to the pre-pandemic period, there was a 31.62% decrease in the number of admitted children during the pandemic period, and a 52.78% reduction in the proportion of respiratory system diseases. The overall mortality rate decreased by 87.81%. There were differences in age and gender distribution between the two periods. The length of hospital stay during the pandemic showed no statistical significance, whereas hospitalization costs exhibited statistical significance.

**Conclusion:**

The COVID-19 pandemic has exerted a certain influence on the disease spectrum of PICU admissions. Implementing relevant measures during the pandemic can help reduce the occurrence of respiratory system diseases in children. Considering the changes in the disease spectrum of critically ill PICU children, future clinical prevention and treatment in PICUs should continue to prioritize the respiratory, neurological, and hematological oncology systems.

## 1 Introduction

The novel coronavirus (coronavirus disease 2019, COVID-19) is characterized by high pathogenicity, high transmissibility, and a high rate of asymptomatic carriers. China has implemented various strategic measures such as advocating mask-wearing, increasing hand hygiene practices, and limiting population movement (online education, home isolation) which have affected the number of hospitalizations and disease patterns among both inpatients and emergency patients (1). Recent studies indicate that patient visits to hospitals decreased significantly after the outbreak of COVID-19, leading to corresponding changes in disease patterns (2–4). Orlei Ribeiro de Araujo et al. (5) found that the coronavirus disease 2019 pandemic strongly affected Brazilian PICUs, reducing admissions, length of stay, and the epidemiological profile. The measures to oppose the coronavirus disease 2019 pandemic may have prevented thousands of PICU hospitalizations across the country. It is widely acknowledged that the reduction in hospital visits could be attributed to the decreased incidence of infectious diseases due to measures aimed at controlling the spread of severe acute respiratory syndrome coronavirus 2 (SARS-CoV-2) (6–10). However, no research has yet explored the impact of the COVID-19 pandemic on the number of cases and disease patterns in the Pediatric Intensive Care Units (PICU) in Hunan Province in China. This study aims to investigate the number of admissions and disease patterns in the PICU of Hunan Children's Hospital during the COVID-19 pandemic period, compare them with pre-pandemic data, analyze relevant changes in disease patterns, and provide guiding insights for pediatric disease prevention and treatment in the field of critical care medicine.

## 2 Data and methods

### 2.1 Study subjects

The study subjects included all inpatients admitted to the PICU of Hunan Children's Hospital between January 1, 2017, and December 31, 2022. The PICU of Hunan Children's Hospital is established on January 5, 1988. It is the National Clinical Key Specialty of People's Republic of China. It currently has 91 medical staff, 20 are doctors, 71 are nurses, 2 of the medical staff have got doctor's degree, and 18 of the medical staff have got Master's degree. And it serves the children from all over Hunan province. Patient information, including gender, age, and disease diagnosis, was collected. In cases with multiple diagnoses, the first diagnosis was used for classification and statistics.

### 2.2 Study methods

Neglecting the initial impact of the pandemic on inpatients, the period from January 1, 2017, to December 31, 2019, was designated as the pre-pandemic group, while the period from January 1, 2020, to December 31, 2022, was designated as the pandemic group. The changing patterns of disease in the two groups were compared. Following commonly used age divisions for children, hospitalized children were categorized into five stages: infancy (29 days to less than 1 year old), toddlerhood (1 to less than 3 years old), preschool age (3 to less than 6 years old), school age (6 to less than 12 years old), and adolescence (12 to less than 18 years old). And ICD-10 was used to collect the diagnosis in the standardized classification system.

### 2.3 Study definitions

We defined improved status as the patient's physical condition has not fully recovered, but has been significantly improved and is gradually developing towards the recovery line of disease. We defined cure status as the patients have been completely cured. We defined uncovered status as the patients have not been completely cured.

### 2.4 Statistical methods

Statistical analyses were performed using the SPSS statistical software program, version 25.0 (IBM Corporation, Armonk, NY, USA). Categorical data were presented as proportions or percentages (%), and comparisons between groups were analyzed using the *χ*^2^ test with a significance level of *α* = 0.05, considering *P* < 0.05 as statistically significant differences. Continuous variables were expressed as median and interquartile range, as normality tested by using normal probability cumulative distribution chart method, was not met. Comparison between groups was thus performed using Mann-Whitney *U*-test.

## 3 Results

### 3.1 Basic characteristics of patients

A total of 8,218 cases were included in the pre-COVID-19 group, and 5,619 cases were included in the post-COVID-19 group. The number of patients in the post-COVID-19 group decreased by 30.12% compared to the pre-COVID-19 group. Among them, 8,621 cases were male children, and 5,216 cases were female, with a male-to-female ratio of 1.65. The gender distribution difference between the two groups was not statistically significant (*P* > 0.05) (Table 1).

**Table 1:** Gender distribution of patients in the Pre-COVID-19 and post-COVID-19 groups.

Group	Male	Female	Total
Pre-COVID-19 group	5,151 (62.68%)	3,067 (37.32%)	8,218
Post-COVID-19 group	3,470 (61.75%)	2,149 (38.25%)	5,619

### 3.2 Age distribution of patients

In the post-pandemic period, the proportion of infant patients was 36.32%, significantly lower than the pre-pandemic period (56.33%) (*P* < 0.01). However, the proportions of preschool, school-age, and adolescent patients increased in the post-pandemic group. Specific age distribution values are shown in Table 2.

**Table 2:** Distribution of patients in the Pre- and post-COVID-19 groups.

Group	Infant	Toddler	Preschool	School	Adolescent
Pre-COVID 19 group	3,909 (47.57%)	2,033 (24.74%)	1,108 (13.48%)	970 (11.80%)	198 (2.41%)
Post-COVID 19 group	2,050 (36.48%)	1,256 (22.35%)	971 (17.28%)	978 (17.41%)	364 (6.48%)

### 3.3 Disease composition by systems before and after the pandemic

**Figure 1:**
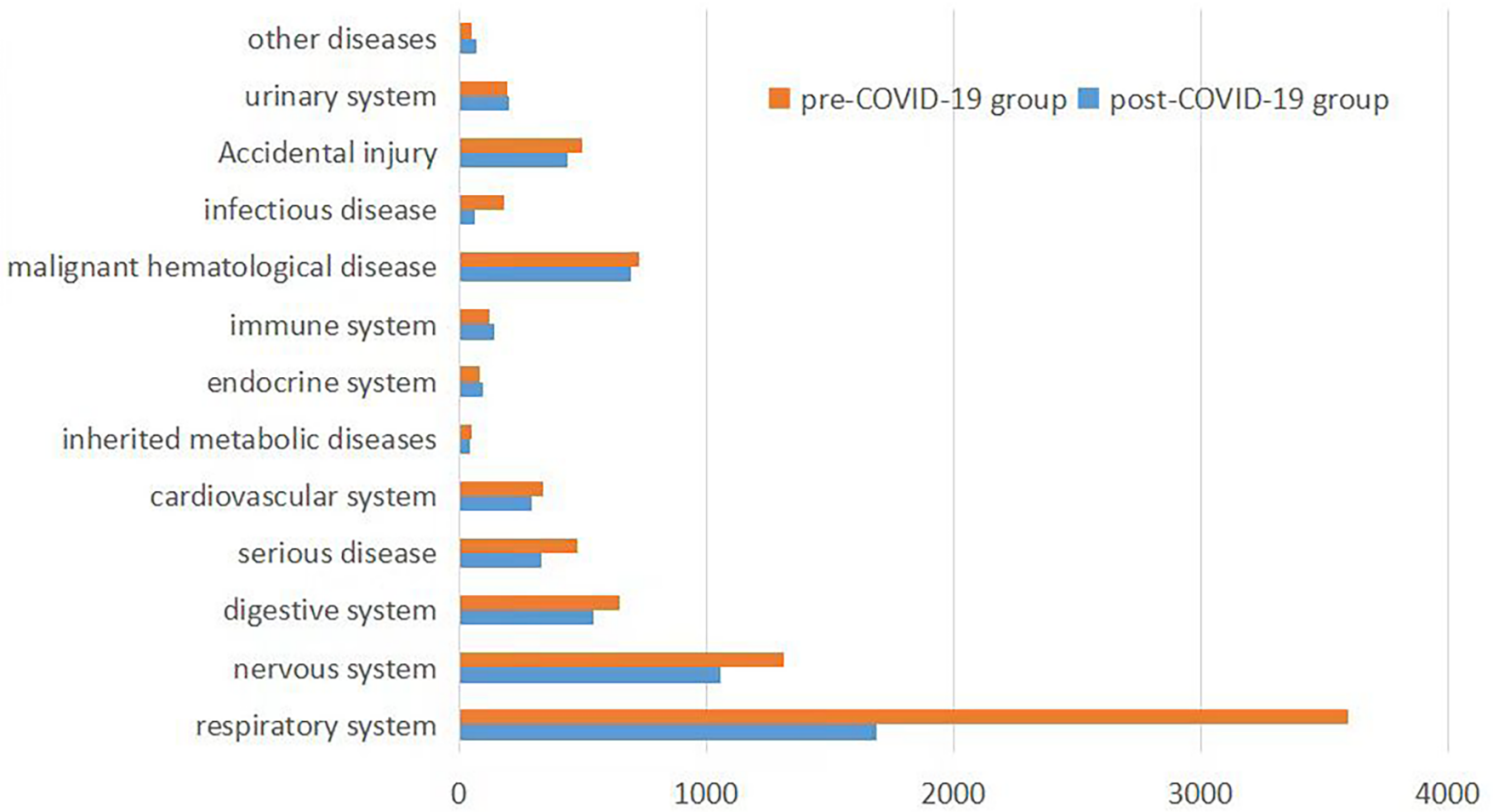
Disease composition of patients in the Pre- and post-COVID-19 groups.

We classified the disease spectrum into respiratory, digestive, nervous, urinary, hematological-oncological, immune, endocrine, critical illness, infectious, genetic-metabolic, cardiovascular, accidental injury, and other systems. The composition of diseases for each system is depicted in Figure 1.

The results reveal that in the pre-COVID-19 group, the top six diseases in the disease spectrum were: (1) Respiratory system diseases (severe pneumonia, acute laryngitis, acute respiratory distress syndrome, etc.), 3,597 cases; (2) Neurological system diseases (epilepsy, viral encephalitis, purulent meningitis, etc.), 1,305 cases; (3) Hematological-oncological system diseases (leukemia, immune thrombocytopenia, hemophagocytic syndrome, etc.), 727 cases; (4) Digestive system diseases (acute gastroenteritis, acute liver failure, etc.), 644 cases; (5) Accidental injuries (poisoning, drowning, car accidents, etc.), 497 cases; (6) Critical illnesses (sepsis, septic shock, organ failure due to various causes, etc.), 470 cases.

In the post-COVID-19 group, the top six diseases in the disease spectrum were: (1) Respiratory system diseases, 1,683 cases; (2) Neurological system diseases, 1,052 cases; (3) Hematological-oncological system diseases, 693 cases; (4) Digestive system diseases, 538 cases; (5) Accidental injuries, 438 cases; (6) Critical illnesses, 326 cases.

### 3.4 Analysis of hospitalization Status before and after the pandemic

**Figure 2:**
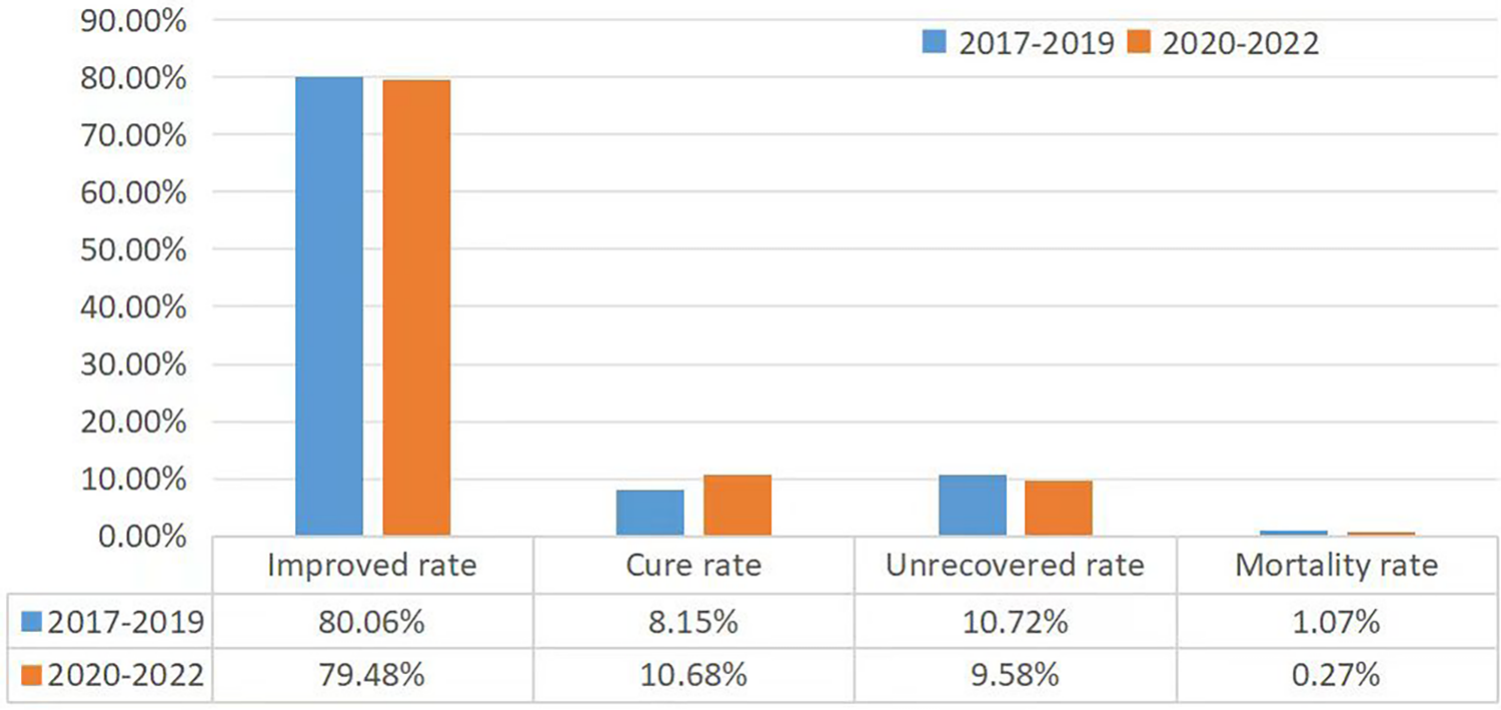
Analysis of disease outcomes in the Pre- and post-COVID-19 groups.

An analysis was performed on the improved rate, cure rate, mortality rate, uncovered rate, average length of hospital stay, and evaluated hospitalization costs for the case data in both groups see Figure 2. In the pre-COVID-19 group, the improved rate is 80.06%, the cure rate is 8.15%, the uncovered rate is 10.72% and the mortality rate is 1.07%. In the post-COVID-19 group, the improved rate is 70.48%, the cure rate is 10.68%, the uncovered rate is 9.58% and the mortality rate is 0.27%. The mortality rate in the PICU during the pandemic control period decreased significantly (*P* < 0.01). The median total length of hospital stay in the pre-COVID-19 group was 12 [8, 20] days, with an average hospitalization cost of 23,775.79 [14,357.42, 43,162.87] yuan. And we calculated the hospitalization costs by using the hospital system, and it is stable in Hunan Province. In the post-COVID-19 group, the median length of hospital stay was 13 [8, 23] days, with an median hospitalization cost of 23,114.49 [13,339.08, 49,074.27] yuan. While there was no statistically significant difference in hospitalization costs, there was a statistically significant difference in hospitalization days, (*P* < 0.05).

## 4 Discussion

### 4.1 Age characteristics

The majority of PICU admissions were concentrated among infants and toddlers, accounting for 72.31% and 58.83% of PICU admissions in the pre- and post-COVID-19 periods, respectively. During the pandemic, the proportion of infants and toddlers decreased, while the proportion of children from preschool to adolescence increased. This decrease in infants and toddlers may be attributed to reduced exposure to the external environment during the pandemic and increased emphasis on personal hygiene and protection due to the pandemic's impact on parents. The increase in the proportion of children from preschool to adolescence might be influenced by negative psychological factors related to the pandemic (11), highlighting the importance of focusing on children's mental health during such periods (12).

### 4.2 Impact analysis of COVID-19 on PICU admissions at Hunan Children's Hospital

The results of this study indicate that while the pandemic did not change the rankings of the top eight disease categories, it significantly influenced the composition of diseases in categories nine to twelve. The COVID-19 pandemic had a certain impact on the disease spectrum of PICU admissions, primarily leading to a significant reduction in the proportion of respiratory system diseases and infectious diseases. The inclusion of three years of data before and after the pandemic, totaling 13,918 cases, reduces the potential error arising from small sample size and strengthens the persuasiveness of this study.

This study revealed a decrease of 53.04% and 66.12% in respiratory system diseases and infectious diseases, respectively. This suggests that the pandemic, while controlling the spread of the novel coronavirus, also curbed the occurrence of respiratory and infectious diseases. Possible reasons include: (1) Government measures to control movement of individuals, adoption of online learning by schools, reduced child outings and gatherings, along with pandemic prevention education such as mask-wearing, increased social distancing, and hand hygiene, led to a decrease in opportunities for cross-infection (13, 14); (2) As the only Grade 3 Children's Specialized Hospital in Hunan Province, our hospital's pre-pandemic inpatients largely comprised patients from other areas. During the pandemic, due to complex treatment procedures and factors like lockdowns, many children with mild symptoms were either observed and treated at home or sought nearby medical care. Simultaneously, the maturation of internet hospitals has led many parents to opt for online consultations, reducing the back-and-forth trips to the hospital and the risk of cross-infection for both patients and their families; (3) Starting from August 2021, Hunan Province progressively initiated the administration of COVID-19 vaccines for students of all ages, in accordance with the province's joint prevention and control mechanism. The COVID-19 vaccine is considered a safe and effective tool to prevent severe infection, hospitalization, and death (15–18). Additionally, the vaccination rate for influenza vaccines has increased significantly compared to pre-pandemic levels, leading to a notable decrease in the incidence of influenza and pneumonia (19).

### 4.3 Impact analysis of COVID-19 on diagnosis and treatment in Hunan Children's Hospital's PICU

Jeng-Hung Wu et al. (20) found that during the COVID-19 epidemic with strict public restrictions, critically ill patients admitted to the PICU decreased but had increased disease severity, prolonged length of stay in the PICU, and higher mortality, reflecting the impact of quarantine and limited medical access. In this study, in the post-COVID-19 period, there was an increase in the average hospitalization cost and an extension of the average length of hospital stay for patients admitted to the PICU of Hunan Children's Hospital. This increase and extension can be attributed to several factors. Most notably, during the pandemic, children with milder conditions often chose home or outpatient treatment instead of seeking inpatient care. Therefore, those who did come to the hospital and required hospitalization tended to have more urgent and critical conditions, often accompanied by complications and comorbidities, making their cases relatively complex. The rise in costs and prolonged hospitalization could also be related to changes in the spectrum of pathogens during the pandemic period.

### 4.4 Study limitation

This is a single center study, and more valuable findings will be found in a nationwide multicenter large sample study. And this is a retrospective study, we don't conduct long-term follow-up on the PICU patients.

## 5 Conclusion

Analyzing the cases admitted to the PICU during the pandemic period provides insight into the changing trends of pediatric disease spectrum. This understanding serves as a scientific basis for improving pediatric medical care and enhancing the quality of critical care for children. During non-pandemic periods, the focus of children's prevention and treatment remains on respiratory system diseases, neurological system diseases, and hematological-oncological diseases.

## Data Availability

The original contributions presented in the study are included in the article/Supplementary Material, further inquiries can be directed to the corresponding author.
